# Novel rRNA-depletion methods for total RNA sequencing and ribosome profiling developed for avian species

**DOI:** 10.1016/j.psj.2021.101321

**Published:** 2021-06-09

**Authors:** Hanwen Gu, Yu H. Sun, Xin Zhiguo Li

**Affiliations:** ⁎ Department of Biomedical Engineering, University of Rochester, Rochester, NY 14642, USA; † Center for RNA Biology, Genome to Therapeutics, Department of Biochemistry and Biophysics, Department of Urology, Department of Biology, Goergen Institute for Data Science, University of Rochester, Rochester, NY 14642, USA

**Keywords:** rRNA, mitochondrial rRNA, chickens, RNA-seq, Ribo-seq

## Abstract

Deep sequencing of RNAs has greatly aided the study of the transcriptome, enabling comprehensive gene expression profiling and the identification of novel transcripts. While messenger RNAs (**mRNAs**) are of the greatest interest in gene expression studies as they encode for proteins, mRNAs make up only 3 to 5% of total RNAs, with the majority comprising ribosomal RNAs (**rRNAs**). Therefore, applications of deep sequencing to RNA face the challenge of how to efficiently enrich mRNA species prior to library construction. Traditional methods extract mRNAs using oligo-dT primers targeting the poly-A tail on mRNAs; however, this approach is not comprehensive as it does not capture mRNAs lacking the poly-A tail or other long non-coding RNAs that we may be interested in. Alternative mRNA enrichment methods deplete rRNAs, but such approaches require species-specific probes and the commercially available kits are costly and have only been developed for a limited number of model organisms. Here, we describe a quick, cost-effective method for depleting rRNAs using custom-designed oligos, using chickens as an example species for probe design. With this optimized protocol, we have not only removed the rRNAs from total RNAs for RNA-seq library construction but also depleted rRNA fragments from ribosome-protected fragments for ribosome profiling. Currently, this is the only rRNA depletion-based method for avian species; this method thus provides a valuable resource for both the scientific community and the poultry industry.

## INTRODUCTION

The high-throughput sequencing of cellular RNAs (**RNA-seq)** or ribosome protected fragments (**Ribo-seq)** has greatly aided in the characterization of the transcriptome, enabling gene expression studies and the identification of novel transcripts and novel open reading frames (Ingolia et al., 2009; Morlan et al., 2012; Adiconis et al., 2013). Protein coding mRNAs are of the greatest interest in gene expression studies. However, efficient RNA deep sequencing faces significant challenges on how to enrich mRNAs in the sample library, as mRNAs only account for 3 to 5% of total RNA (Alberts et al., 2002). Therefore, applications of high-throughput sequencing approach to RNAs require methods for enriching mRNAs.

One widely-used method for mRNA enrichment uses oligo dTs to capture the mRNAs with poly-A tails. However, this approach is incapable of capturing mRNAs that are missing poly-A tails due to degradation, or mRNAs such as histone mRNAs that use alternative mechanisms for 3´ (primer) end processing (López and Samuelsson, 2008). An alternative method involves the depletion of ribosomal RNAs (**rRNAs**). Because ribosomes are required for protein synthesis in the cytoplasm and mitochondria, rRNAs are abundant, accounting for ~80% of total RNA in eukaryotes (Lodish et al., 2000). Cytosolic rRNAs, including 18S, 28S, 5.8S, and 5S rRNAs, are processed from 2 rRNA precursors, and the general scheme of their processing is conserved among mammals, invertebrates, yeast, and plants (Long and Dawid, 1980; Tomecki et al., 2017). One of the rRNA precursors, the 45S rRNA precursor, is transcribed by RNA polymerase I, and then further processed into 18S, 5.8S, and 28S (Winnebeck et al., 2010). The second rRNA precursor, the 5S precursor, is transcribed by RNA polymerase III (Winnebeck et al., 2010). Finally, all mature rRNAs are assembled with ribosomal proteins to form functional small and large subunits. Distinct from cytosolic rRNAs, mitochondria rRNAs, 12S and 16S rRNAs, are encoded by the mitochondrial genome (Rorbach and Minczuk, 2012), transcribed and processed within mitochondria, and further assembled with ribosome subunits. These mitochondrial rRNAs are responsible for ribosome functions inside mitochondria (Yang et al., 2014).

The depletion of rRNAs is not only a preferred mRNA enrichment method for RNA-seq, but also the only option for Ribo-seq. Ribo-seq is a technique to capture ribosome protected fragments (**RPFs**) on a genome-wide scale, with a sub-codon resolution (Ingolia et al., 2009). RPFs are acquired by treating tissue/cell lysate with an optimized amount of RNase, which degrades the RNAs that are not protected by ribosomes. After running a sucrose gradient, the monosome fraction will be collected to enrich authentic RPFs. Since rRNA is a significant component of ribosomes, the purified RNAs from sucrose gradient also have an abundance of rRNA. Thus, it is also necessary to deplete rRNA before RPF library construction. rRNA-depletion approaches typically use oligos complementary to rRNAs to either extract them or degrade them from the total RNA sample. A major limitation of such antisense oligo-based approaches, however, is that they require species-specific probes. Thus, the commercially available kits are not only costly and but also have been limited to a small number of model organisms, not including chickens.

The chicken has been widely-used for studying various biological questions. Chickens have a sex-determination system different from that of most mammals, wherein males are the homogametic sex (ZZ) and females are the heterogametic sex (ZW). Chicken primordial germ cells (**cPGCs**) are currently the only type of PGCs that can be cultured in vitro for long term (Park and Han, 2012), providing a unique opportunity for extensive biochemical studies, including genetic manipulation (Naito et al., 2015; Han and Lee, 2017). Studying chickens benefits not only basic research but also the poultry industry. Chicken is one of the most significant food sources in the world, with over 50 billion chickens being consumed every year (Boyd and Watts, 2013). Furthermore, given the adaptive radiation of birds (over 10,000 species), chickens are valuable models for endangered avian species conservation (Muir and Aggrey, 2003). Investigating chicken genomes and transcriptomes may, therefore, shed light on avian evolutionary processes and contribute to conservation biology (Li et al., 2019).

Here we focus on developing a method for depleting rRNAs from total RNA using oligos specially designed for chickens. These oligos are antisense to cytosolic and mitochondrial rRNAs, and the resulting DNA-RNA hybrids can be removed using RNase H. RNase H is an endoribonuclease that recognizes DNA-RNA hybrid molecules and hydrolyzes the RNA strand (Inoue et al., 1987). DNase is then used to digest the remaining DNA in the mixture. We have succeeded in using this protocol not only to remove rRNAs from total RNAs for RNA-seq library construction, but also to remove rRNA fragments for Ribo-seq. Thus, our method advances previously developed rRNA depletion method (Culviner et al., 2020) in the following 2 aspects: 1) significantly benefits the avian research community, and 2) with broader applications for both RNA-seq and Ribo-seq studies.

Every step of the protocol has been optimized. Specifically, we optimized the ratio between RNAs and oligo probe, the ideal brand of RNase H enzyme, the amount of RNase H, as well as the treatment time and temperature. For total RNA, we monitored our depletion efficiency by the ratio of GAPDH to rRNAs and the success of depletion is shown by bioanalyzer profiles. For RPFs, we adopted the optimized condition from total RNA rRNA depletion, but further optimized the temperature of RNase H treatment as the annealing differs by fragment length. We monitored the depletion efficiency by Ribo-seq and the ratio of rRNA reads in the library (Table 1). Our strategy has been successfully applied to chickens, and we envision no problems in applying it to other species.

**Table 1:** Read statistics of Ribo-seq libraries constructed with different treatment temperatures.

Name	Total reads	rRNA mapping reads	% rRNA mapping reads
Sample 1 - 30°C	763,350	372,204	48.8
Sample 2 - 37°C	165,239	63,454	38.4
Sample 3 - 42°C	743,014	301,481	40.6
Sample 4 - 45°C	937,034	388,145	41.4
Sample 5 - Room Temperature	973,777	475,268	48.8

Our protocol is cost-effective and, to the best of our knowledge, is currently the only rRNA depletion-based method for avian species, thus providing a valuable resource for the community. Here, we outline the steps for extracting total RNA from animal tissue, depleting rRNAs, and purifying the enriched mRNA sample.

## MATERIALS AND METHODS

**Figure 1:**
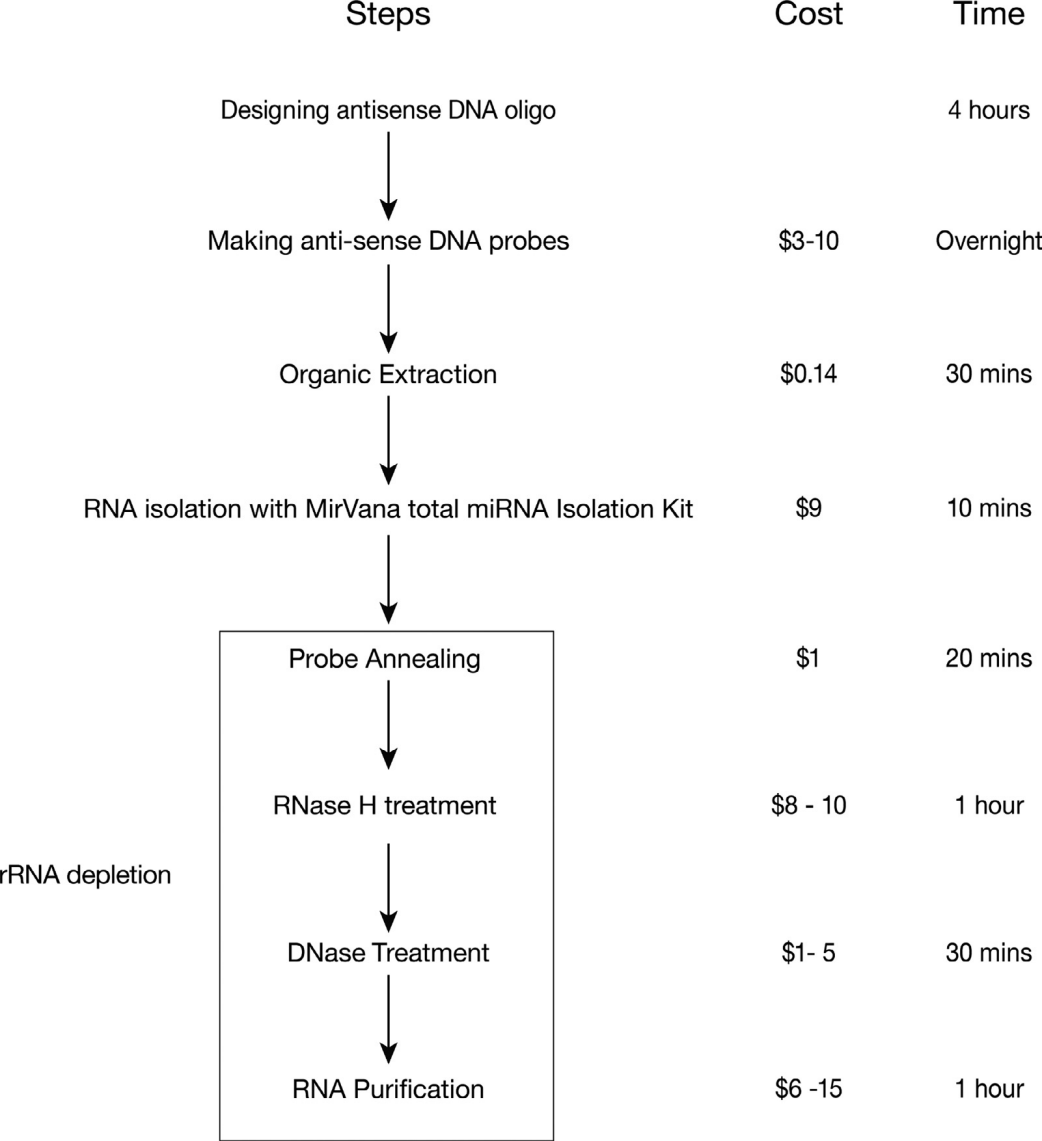
A flowchart summarizing the protocol.

The RNA depletion protocol outlined here consists of 2 parts: the oligo-based rRNA depletion (Figure 1) and the bioinformatic analysis of the result (https://github.com/LiLabZhaohua/RiboSeqPipeline).

### Designing the Antisense Oligo

To design the antisense oligo, we downloaded the rRNA sequences of both cytosolic ribosomes and mitochondrial ribosomes in chicken species from NCBI. These are mitochondrial rRNAs including the 16S rRNA (XR_003076910.1), and 12S rRNA (MH879470), and cytosolic rRNAs which for eukaryotes include 18S rRNA (AF173612.1), 28S rRNA (XR_003078040.1), 5.8S rRNA (XR_003078039.1) and 5S rRNA (XR_003076910.1). In order to minimize the chances of mismatch, for species not close related to chicken, a specific set of oligos should be designed and synthesized according the following steps.

DNA oligos were designed by converting the original sequences to their reverse complements and splitting the full-length sequences into 50 nt non-overlapping windows using a python script [https://github.com/LiLabZhaohua/rRNADepletion].

• To minimize potential oligo mismatch pairing with other RNAs, the basic local alignment tool (**BLAST**) was used to find possible pairing with other RNAs in chickens. We confirmed that these oligos do not have significant targets in the chicken transcriptome (Supplementary Table 1).
• A pool of DNA probes was made, after receiving the oligos synthesized by Integrated DNA Technologies (Coralville, IA), by mixing the 5.8S, 28S, 18S, 16S, 12S, and 5S oligos to a final concentration of 0.5 μM for each oligo.

### Total RNA Isolation

This step is done with the mirVana total miRNA isolation kit (cat. AM1560; Thermo Fisher, Waltham, MA), but any other total RNA isolation kits can be applied to this step.

#### Frozen Tissue or Extremely Hard Tissues

• Remove the tissue from the −80°C freezer.
• Cut out 25 mg of frozen tissue and place into a 1.7 uL tube.
• Add 300 uL Lysis/Binding Buffer into a plastic weigh boat or tube on ice.
• Grind frozen tissue into a powder using a homogenizer.
• Add 30 uL miRNA Homogenate Additive to a tube. (Note: It is important to add the Homogenate additive immediately to the tube after the tissue is ground as it prevents the degradation of RNA.)
• Leave mixture on a rotator for 10 min.

#### Organic Extraction

Add 300 uL of acid chloroform (Thermo Fisher cat. AM9722). Withdraw the bottom layer, avoiding the upper phase as this is mostly aqueous buffer.

• Vortex the mixture for 60 s to mix well.
• Centrifuge the mixture for 5 min at 16,900 RCF at room temperature. (Note: The separation between the top and bottom phase should be clear. If the interphase is not compact, repeat the centrifugation process.)
• Transfer the upper layer to a new 1.7 uL tube. It should be approximately 300 uL. It is very important to not disturb the bottom phase since it is mostly tissue and acid phenol.

#### RNA Isolation Procedure

Wash solution 1 and solution 2/3 should be pre-mixed with ethanol according to the manual. At the end of the final stage, RNA can be eluted with either nuclease-free water or in the Elution buffer provided by the kit.

• Add 375 uL of room temperature 100% ethanol to the aqueous phase.
• Place the column on a collection tube.
• Pipet the mixture to the column.
• Centrifuge for 30 s at 9,300 RCF.
• Discard the flow through.
• Add 700 uL miRNA wash solution 1 (Premixed with ethanol).
• Centrifuge for 10 s.
• Discard the flow through.
• Add 500 uL miRNA wash solution 2/3 (Premixed with ethanol).
• Centrifuge for 10 s.
• Discard the flow through.
• Add 500 uL miRNA wash solution 2/3 (premixed with ethanol).
• Centrifuge for 10 s.
• Discard the flow through.
• Centrifuge for another 3 min to remove the liquid residual.
• Preheat the nuclease-free water to 95°C.
• Transfer the column into a new collection tube.
• Add 100 uL preheated elution buffer or nuclease-free water.
• Spin for 4 min at 11,300 RCF at room temperature.
• Collect the elutes and store it at −80 °C.

### rRNA Depletion

For this step, the enrichment for mRNAs and RPFs follows the same steps except for the temperature for RNase H treatment and the last step, in which the depletion from total RNAs uses the RNA Clean & Concentrator kit (cat. R1013; ZYMO Research, Irvine, CA) protocol, while the depletion from RPF uses the acid phenol protocol.

#### Depletion Preparation

In this step, we prepare 3 solutions that will be used for rRNA depletion later.

For the probe oligo, prepare the 5X rRNA oligo hybridization buffer and 10X RNase H and aliquot for use.

Prepare 5X rRNA oligo hybridization buffer: mix 100 mM Tris-HCl with 200 mM NaCl.

Prepare 10X RNase H digestion buffer: mix 500 mM Tris-HCl with 1 M NaCl and 200 mM MgCl_2_.

#### rRNA Depletion From Total RNA

##### Probe Annealing

• Pipette 2 μg of RNA and 15 μL DNA oligo probe into a tube, adjusting the volume of 5X rRNA oligo hybridization buffer and water according to Table 2.

**Table 2:** Probe annealing system.

Name	System 1	System 2	System 3
RNA	2 ug	2 ug	2 ug
5X rRNA oligo hybridization buffer	5 uL	6 uL	7 uL
DNA oligo probe	15 uL	15 uL	15 uL
ddH_2_O	to 25 uL	to 30 uL	to 35 uL
• You may adjust the volume and choose one system in Table 2, or use a larger total volume, but a total volume over 45 μL is not suggested.
• You may also dilute the system, using the 1X rRNA oligo Hybridization Buffer, after you mix all the components (not over 45 μL for 2μg RNA).
• Use the PCR machine for annealing: Heat to 95°C for 2 min and slowly cool down (−0.1 °C/sec) to 22°C, then incubate at 22°C for additional 5 min.

##### RNase H Treatment

• Turn on the PCR machine and set the temperature to 45°C.
• Add the solutions and Invitrogen RNase H (Thermo Fisher cat. 18021071) listed in Table 3, according to the volumes of annealing.

**Table 3:** RNase H treatment system.

Name	System 1	System 2	System 3
10X RNase H digestion buffer	3 uL	3.5 uL	4 uL
Invitrogen RNase H	1 uL	1 uL	1 uL
ddH_2_O	1 uL	0.5 uL	0 uL
Total volume	30 uL	35 uL	40 uL
• Incubate at 45°C for 1 h in the PCR machine.

##### DNase Treatment

• Take out samples from the PCR machine and adjust the PCR machine to 37°C.
• Immediately add the components listed in Table 4. Note that regardless of which system you chose, the total volume should be 50 uL.

**Table 4:** DNase treatment system.

Name	System 1	System 2	System 3
10X Turbo DNase buffer	5 uL	5 uL	5 uL
Turbo DNase	2 uL	2 uL	2 uL
ddH_2_O	13 uL	8 uL	3 uL
Total volume	50 uL	50 uL	50 uL
• Incubate at 37°C for 30 min in the PCR machine.

##### RNA Purification

This step uses the RNA Clean & Concentrator kit (Thermo Fisher cat. R1013) protocol. Prepare adjusted RNA Binding buffer by mixing equal volumes of buffer and ethanol.

• Transfer 50 uL of RNA to a new 1.7 mL tube.
• Add 50 uL water to the tube.
• Add 200 uL adjusted binding buffer to the tube.
• Transfer the mixture into a column.
• Centrifuge for 30 s at 16,900 RCF at room temperature.
• Discard the flow-through.
• Add 200 uL RNA prewash.
• Centrifuge for 30 s at 16,900 RCF at room temperature.
• Discard the flow-through.
• Add 200 uL RNA wash buffer.
• Centrifuge for 30 s at 16,900 RCF at room temperature.
• Discard the flow-through.
• Add 200 uL RNA wash buffer.
• Centrifuge for 2 min at 16,900 RCF at room temperature.
• Discard the flow-through.
• Transfer the columns into new 1.5 mL tubes (RNase Free tube).
• Add 15 uL DNase/RNase-free water directly to the column matrix.
• Let the columns sit at room temperature for at least 1 min.
• Centrifuge for 30 s at 16,900 RCF at room temperature.
• Keep the flow through in the tube and store the tube at −80°C immediately.

#### rRNA Depletion From RPFs

##### Probe Annealing

• Pipette 2 μg of RNA and 15 μL DNA oligo probe into a 1.7 mL tube.
• Adjust the volume of 5X rRNA oligo hybridization buffer and water according to Table 2.
• You may adjust the volume and choose one system in Table 2, or use a larger total volume, but a total volume over 45 μL is not suggested.
• You may also dilute the system, using the 1X rRNA oligo hybridization buffer, after you mix all the components (not over 45 μL for 2μg RNA).
• Use the PCR machine rRNA program for annealing: Heat to 95°C for 2 min and slowly cool down (−0.1°C/sec) to 22°C, then incubate at 22°C for additional 5 min.

##### RNase H Treatment

• Turn on the PCR machine and set the temperature as 37 °C for RPF for optimal results. * We have compared the treatment temperature at room temperature, 30°C, 37°C, 42°C, and 45°C, and found that 37 °C has the best removal based on our sequencing results (Table 1).
• Add the solutions and RNase H (Thermo Fisher cat. 18021071) listed in Table 3, according to the volumes of annealing.
• Incubate at 37°C for 1 h on PCR machine.

##### DNase Treatment

• Remove the samples from PCR machine and adjust the PCR machine to 37°C.
• Immediately add the components listed in Table 4. (Note: The total volumes should all be 50 uL.)
• Incubate at 37°C for 30 min in the PCR machine.

##### Purification of RPF

• Transfer 50 uL RNA to a 1.7 mL tube.
• Add 200 μL ddH_2_O.
• Add 300 μL acid phenol chloroform (Thermo Fisher cat. AM9722). Note the total volume should be around 550 uL.
• Vortex for 1 min to mix well.
• Spin at 20,100 RCF for 15 min at room temperature.
• Withdraw the upper phase (around 250 uL) without disturbing the lower phase and transfer the solution to a new 1.7 mL tube.
• Add 1 μL glycogen (cat. 10901393001; Sigma Aldrich, St. Louis, MO).
• Add 3 volumes (around 750 uL) of 100% ethanol.
• Leave at 4°C for an hour, or −20°C overnight. White precipitate should be visible.
• Spin down at 16,900 RCF for 30 min at 4°C.
• Wash with 200 uL of 70% ethanol.
• Centrifuge at 16,900 RCF for 5 min at highest speed at 4°C.
• Pour out the liquid.
• Centrifuge at 16,900 RCF for another 3 min at highest speed at 4°C.
• Pipette out all the liquid and leave only the pellet in the tube.
• Dissolve in water at room temperature.
• Store at −80°C Table 5, Table 6.

### Data Analysis

We analyzed the Ribo-seq as previously described by Sun et al. (2020). Ribo-seq pipeline for chicken was developed for this study, which is available in the following link (https://github.com/LiLabZhaohua/RiboSeqPipeline). Before genome mapping, this pipeline automatically maps Ribo-seq reads to 5S and 45S ribosomal RNA and count the rRNA reads. The rRNA percentage was calculated by dividing rRNA mapping reads to total genome mapping reads. A Student's *t* test was performed between our method with another method in R 3.5.0 using *t* test function, with alternative = "less" parameter. 12S rRNA depletion efficiency was measured by RT-qPCR and analyzed using DART-PCR (Peirson et al., 2003).

## RESULTS

In this protocol, we describe a simple method for enriching mRNAs from animal tissue using an rRNA depletion-based approach. Each key step of the protocol reported above has been optimized, including the ratio between RNAs and oligo probes, the ideal brand of RNase H enzyme, the amount of RNase H, as well as the treatment time and temperature. This is, to the best of our knowledge, the first rRNA depletion method developed for avian species. Although the probes are designed to target chicken rRNAs, considering the conservation of rRNA sequences, we expect that our probes can be used for diverse avian species.

For total RNAs, we used the ratio of 28S to GAPDH to measure the depletion efficiency. We first compared efficiency of 3 commercial RNase H enzymes – RNase H (NEB cat. M0297S) and thermostable RNase H (cat. M0523S; New England Bio Labs, Ipswich, MA) from NEB, and RNase (Thermo Fisher cat. 18021071) from Invitrogen with 1 uL probe, 1 uL RNase H treated for 30 min at 37°C and 45°C. The smallest Relative quality value with respect to the control value for RNase H from Invitrogen indicates the best result at 37°C. Then we compared the Invitrogen RNase H and NEB RNase H with 1 uL and 2 uL at 37°C and 45°C with 3 μL probe for 1 h of RNase treatment and then 30 min of DNase treatment. The result indicates that Invitrogen has the best efficiency at 45°C. After the optimal enzyme chosen, the optimal amount of probe was also investigated with 1μL Invitrogen RNase Treatment at 45°C for 1 h and then DNase treatment for 30 min. Different ratios of the RNA and probe, including 1:0, 1:1, 1:2, and 1:3 were tested. In the end, the optimal condition for RNA depletion is 1 μg RNA: 3 μL probe, 0.2 μL Invitrogen RNase H treatment at 45°C for 1 h, and DNase treatment at 37°C for 30 min. The result was expressed as the ratio of 28S to GAPDH, decreased >4,700 fold from 706 to 0.15 after rRNA depletion, measured by RT-qPCR.

**Figure 2:**
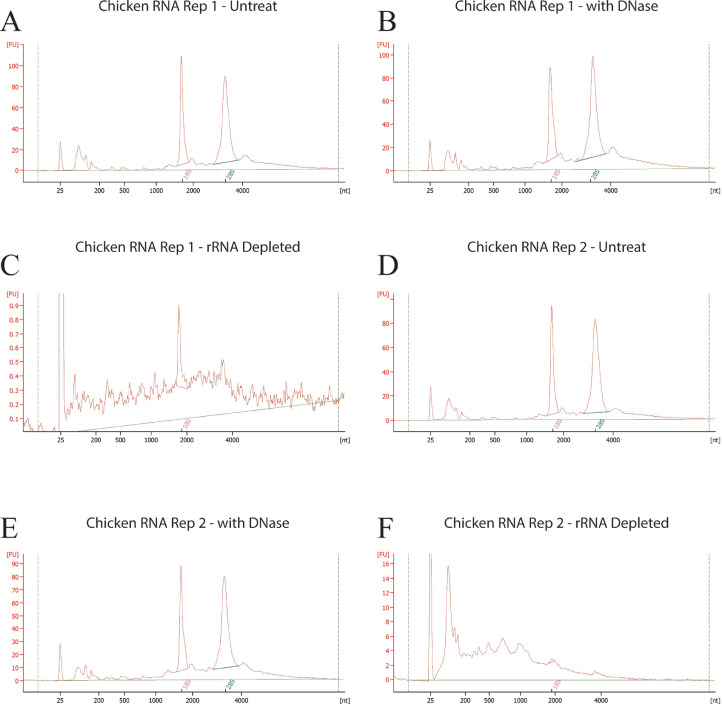
Total RNA bioanalyzer profile, where x axis is RNA size in nucleotides and y axis is the fluorescence unit. Panel A-C, D-F represent untreated, DNase treated and rRNA depleted profiles of two individual chicken RNA replicates. The profile is typically expressed with 2 major peaks corresponding to the 18S and 28S ribosomal RNAs (A, D).

The 260 of 230 values for "pure" nucleic acid are often used to examine whether contamination such as salt or protein exists in the sample or to measure the purity of nucleic acid before and after. Expected 260 of 230 values are commonly in the range of 2.0 to 2.2. If the ratio is appreciably lower, it may indicate the presence of contaminants which absorb at 230 nm. The ratio would be decrease largely due loss of rRNA. As shown in Figures 2A–2F, 2 sets of replicates that have a stable 260/230 ratio before (Figures 2A and 2D), and after which are considered successfully treated with DNase (Figure 2B and 2E).

The 260 of 280 values indicate the purity of RNA. A ratio of ~2.0 is generally accepted as "pure,” while pure DNA has a ratio close to 1.8. Figures 2A, 2D and Figures 2B, 2E are 2 sets of replicates that have a stable 260/280 ratio before and after DNase treatment. This result reveals the successful removal of rDNA oligos.

As expected, we observed a significant decrease of RNA amount after depletion, as 90% of the RNA, which is ribosomal RNA, is now depleted. The decrease of the RNA concentration indicates that Figures 2C and 2F are 2 successful treatments.

The success of rRNA depletion is also demonstrated by the bioanalyzer results. The RNA integrity number (**RIN**) is an index for assigning integrity values to RNA measurements. No significant change should be observed for a successful experiment before and after DNase treatment. Figures 2A, 2B and Figures 2D, 2E are 2 sets of replicates that have a stable RIN before and after DNase treatment indicating the success of the treatment. From the bioanalyzer profiles (Figure 2), 18S and 28S rRNA peaks disappeared after depletion at the optimal condition which is 1 μg RNA:3 μL probe, with 0.2 μL Invitrogen RNase treatment at 45°C for 1 h, and then DNase treatment at 37°C for 30 min. The experiment is considered successful when all ribosomal RNA, including 18S and 28S, are depleted by the end of the procedure. The lack of any rRNA peaks demonstrates the complete removal of rRNA in the rRNA-depletion process (Figures 2B and 2E are 2 replicates of the samples after depletion). The small peaks that can be seen typically correspond to some small RNA and mRNA.

For Ribo-seq, we used the percentage of rRNA fragment in the sequencing results to measure the depletion efficiency. Because the RNA length differs between full-length mRNAs and RPFs, likely to affect the annealing temperature, we therefore constructed Ribo-seq library with the RNase H treatment temperature at RT, 30°C, 37°C, 42°C, and 45°C, respectively. Table 1 listed the read statistics of the Ribo-seq libraries showing the number of total reads and number of reads mapping to the rRNA sequences. Based on the result in Table 1, the optimal result of RPF depletion is seen at 37°C with the lowest percentage of reads coming from rRNAs. Therefore, 37°C is used for RPF depletion protocol.

Additionally, we have included three sets of data (GSM752567, GSM752568, GSM1064855) as a comparison to the poly-A selection results from published papers (Brawandet al., 2011; Necsulea et al., 2014). By running the pipeline we developed, we quantified 5S and 45S rRNA reads and displayed in Table 7, with three replicates for each method. Our method which has an average rRNA percentage of 0.1% in total genome mapping reads, is significantly better than the commercial rRNA-depletion kit which has an average rRNA percentage of 1.5% in total genome mapping reads (Student's *t* test *P* = 0.02). Our method also outperforms poly-A selection method, which has an average rRNA percentage of 2.1% (Student's *t* test *P* = 0.02). We also compared the depletion performance on mitochondria rRNAs. Using another internal control, *ACTB*, our method has an average 22.67 times decrease of 12S comparing to the untreated sample, which is slightly better than the poly-A selection method, with an average of 19.11 times decrease (Student's *t* test *P* = 0.22) (Table 8).

**Figure 3:**
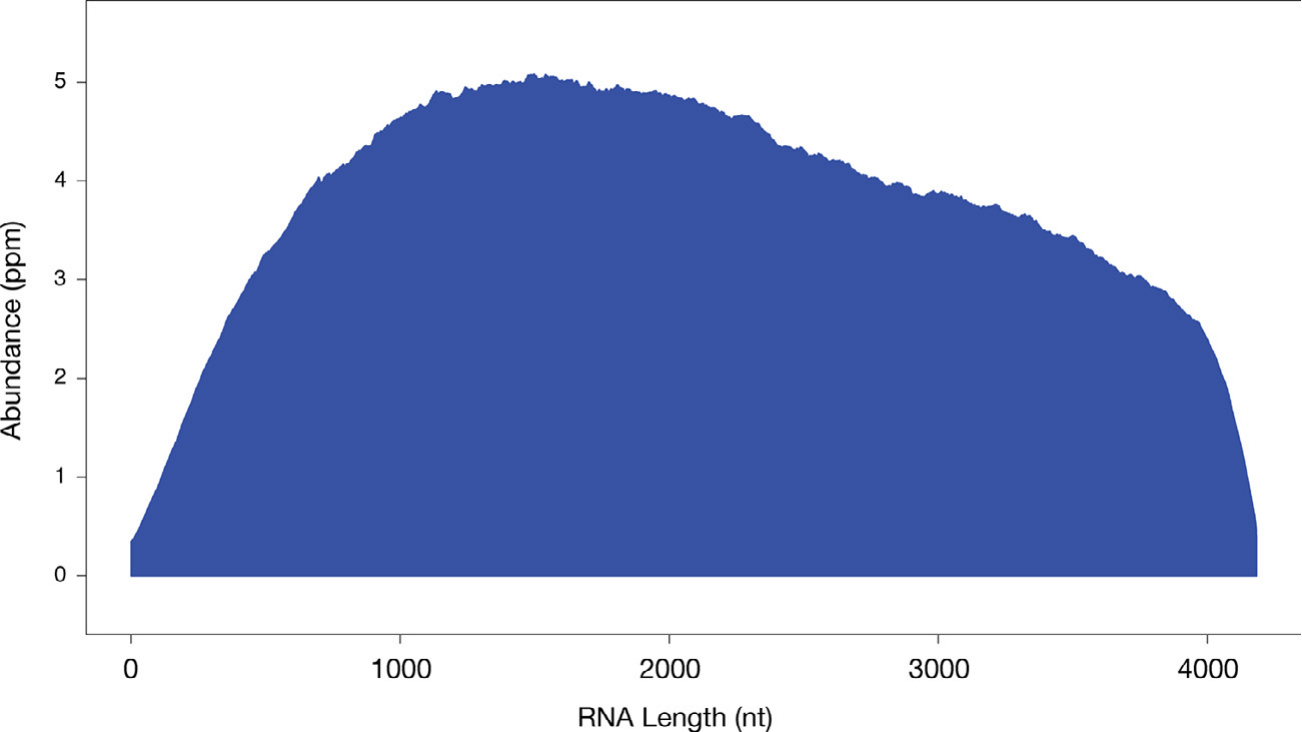
Metagene plots of RNA-seq at chicken mRNAs. The x-axis shows the median length of all chicken mRNAs, and the y-axis represents the ppm (parts per million).

Our results are consistent with what have been reported in mice (Adiconis et al., 2013), which indicates the RNase H method is robust and capable of depleting rRNAs from low input samples. As described before (Adiconis et al., 2013) that compared to a commercial rRNA depletion method, this method has a significantly higher efficiency in rRNA removal (Table 7, Student's *t* test *P* = 0.02), we also found the chicken RNA-seq signals evenly distribute throughout the mRNAs as shown by the metagene analysis of a chicken RNA-seq results (Figure 3). This result indicates that our method won't alter RNA-seq reads distribution across mRNAs, and thus represent

## DISCUSSION

The RNase H method is robust and capable of depleting rRNAs from low input samples (Adiconis et al., 2013). Compared to a commercial rRNA depletion method, this method has a significantly higher efficiency in rRNA removal (Adiconis et al., 2013) and the RNAseq reads distribute normally by the metagene analysis (Figure 3).

The whole procedure takes about 2.5 h starting with purified RNA, and 3.5 h starting with organic tissue. The procedure time is comparable to those of traditional methods, which take approximately 2 h. The price of the method of interest would vary from $20 per reaction starting with total RNA (as seen in Table 5) to $29 starting with organic tissues (seen in Table 6), while the cost of traditional methods varies from $55 to $118. By comparing the price and time with other traditional mRNA purification methods, as seen in Table 6, our method is cost-effective and efficient, while maintaining an excellent quality of purification.

**Table 5:** Cost estimation of the method of interest.

Name of material/equipment	Quantity	Company	Cat. #	Cost
DNA Oligo	32 mL	Integrated DNA Technologies, Coralville, IA	N/A	$912, $5 per reaction
TURBO DNase(2 U/uL)	5000 units	Thermo Fisher, Waltham, MA	AM2239	$309, $0.24 per reaction
RNase H (2 U/µL)	120 units	Thermo Fisher (Invitrogen)	18021071	$489, $8.2 per reaction
RNA Clean & Concentrator Kits	50 prep	ZYMO Research, Irvine, CA	R1013	$170, $3.4 per reaction
Acid phenol chloroform	400 mL	Thermo Fisher, Waltham, MA	AM9722	$197, $0.14 per reaction
Glycogen	1 mL	Sigma Aldrich, St. Louis, MO	10901393001	$151, $3.02 per reaction
Total cost estimation				$2,228 Total $20 per reaction

**Table 6:** Other methods.

Name	Amount	Company	Cat. #	Cost
TruSeq stranded total RNA	48 prep	Illumina, San Diego, CA	20020596	$5660 Total, $118 per reaction
SMART stranded RNA-Seq Kit	48 prep	Takara, Kusatsu, Shiga, Japan	634838	$2920 Total, $60 per reaction
RiboMinus human/mouse transcriptome isolation kit	6 preps	Thermo Fisher, Waltham, MA	K155001	$600, $100 per reaction
NEBNext rRNA depletion kit (Human/Mouse/Rat)	6 preps	New England Bio Labs, Ipswich, MA	E6310S	$330, $55 per reaction
Dynabeads mRNA DIRECT kit (poly-A method)	20 preps	Invitrogen, Waltham, MA	61011	$768, $38.4 per reaction

**Table 7:** A comparison of different rRNA removal strategies.

Method type	Replicate	rRNA/total genome mapping	Mean	Stdev	*t* test *P* value
Commercial rRNA depletion	rep1	0.895%	1.5%	0.5%	0.02
Commercial rRNA depletion	rep2	1.742%			
Commercial rRNA depletion	rep3	1.872%			
Our method	rep1	0.071%	0.1%	0.1%	NA
Our method	rep2	0.128%			
Our method	rep3	0.202%			
Poly-A selection	rep1	0.728%	2.1%	2.7%	0.02
Poly-A selection	rep2	5.229%			
Poly-A selection	rep3	0.389%

**Table 8:** A comparison of 12S rRNA removal using qPCR.

Method type	Replicate	rRNA depletion efficiency	Mean	Stdev	*t* test *P* value
Poly-A selection	rep1	12.0886459	19	10	0.22
Poly-A selection	rep2	26.1417718			
Our method	rep1	20.9366223	33	17	
Our method	rep2	44.5483418			

Furthermore, we aligned the 18S rRNA sequences from four avian species, including chicken (*Gallus gallus*), zebra finch (*Taeniopygia guttata*), north island brown kiwi (*Apteryx mantelli*) and turkey (*Meleagris gallopavo*). As shown in Supplementary Figure 1, these avian species share a high similarity of 18S sequences, indicating a broad application of our oligos in other *Aves* species. Depending on how the oligos are designed and which type of sequence the oligos are specified to target, the same method can potentially be applied for a wide range of organisms and different types of RNA. As long as the oligo is designed to antisense to the specific type of RNA of a specific species, this method should guarantee good quality of purification.

## Appendix Supplementary materials

Image, application 1

Image, image 2

**Supplementary Figure 1:** Alignment of 18S sequences from four avian species: chicken (*Gallus gallus*), zebra finch (*Taeniopygia guttata*), north island brown kiwi (*Apteryx mantelli*) and turkey (*Meleagris gallopavo*). Image, image 3

## Footnotes

Supplementary material associated with this article can be found in the online version at doi:10.1016/j.psj.2021.101321.
