# Supplementary material for: Novel rRNA-depletion methods for total RNA sequencing and ribosome profiling developed for avian species
Source: Poult Sci. 2021 Jun 9;100(9):101321. doi: 10.1016/j.psj.2021.101321 (PMC8322463; doi:10.1016/j.psj.2021.101321)
Supplement: Supplementary file 1 [file mmc1.docx]

**Supplementary Files:**

Supplementary Table I: Sequence used for constructing Probe

| 5.8S_0 | AGCTGCGTTCTTCATCGACGCACGAGCCGAGTGATCCACCGCTAAGAGTT |
| --- | --- |
| 5.8S_50 | GAAGTGTCGATGATCAATGTGTCCTGCAATTCACATTAATTCTCGCAGCT |
| 5.8S_100 | CGCTCAGGCAGGCGTAGCCCCGGGAGGAACCCGGGGCCGCAAGTGCGTTC |
| 28S_0 | CTGACTAATATGCTTAAATTCAGCGGGTCGCCACGTCTGACCTGAGGTCG |
| 28S_50 | CTCTTCACTCGCCGTTACTGAGGGAATCCTCGTTAGTTTCTTTTCCTCCG |
| 28S_100 | ACACCTCCCGCGCCCCACCGCGGGGCGGGGATTCGGCGCTGGGCTCTTCC |
| 28S_150 | GGACTTGGGCCCCCCGAGAGCGGCGCCGGGGATGGGGGCTTCCGTACGCC |
| 28S_200 | GGGGGCCGCTACCGGCCTCACACCGTCCGCGGGCTGGGCCTCGATCAGAA |
| 28S_250 | CTGCATTCCCAAGCAACCCGACTCCGAGAAGCCCCGGGCCCGGCGCGCCG |
| 28S_300 | GGTCTCGTGCCGGTATTTAGCCTTAGATGGAGTTTACCACCCGCTTTGGG |
| 28S_350 | CTCTCTTCAAAGTTCTTTTCAACTTTCCCTTACGGTACTTGTTGGCTATC |
| 28S_400 | GCGCGGACCCCACCCGTTTACCTCTTAACGGTTTCACGCCCTCTTGAACT |
| 28S_450 | GCCCGCGCCGGCCGACCTTGGCCCGCCGGGTTGAATCCTCCGGGCCGACT |
| 28S_500 | CCCGGCGAAGGGGAGGGACGGAGGGGAGGCGGAGGCGGGGATCCGACGGC |
| 28S_550 | CGGCCGGGCGGCGGTCCCCGGCCCGCCCGCCCCCCCTGGGCCCGCCCCGC |
| 28S_600 | GGTCGCGGCGCACCGCCGCGGAGGAAATGCGCCCGACGGGGGCCGGACGC |
| 28S_650 | GGCGCCGGGCCACCTGCCCGCCGGCAGCCCTTCCCAGCCGTCCCGGAGCC |
| 28S_700 | TCGGCGACGATCCGGGCCCGGCGGCTATAACACCCGGCGGCCGCTCGCGC |
| 28S_750 | CCCCCTCCGGGGGAGGGCGCGGCGGCGGTCCTCTCCCTCGGCCCCGGGAT |
| 28S_800 | CGCGGCCGGCCCGACGCCGGTCCGGCCGCGGGGGGCCCTCCGGGGGGCCG |
| 28S_850 | GGAGAGAGAGCGAGCGGCGGCGTACGGCGGCGGCGCGGACGCGCGCGCGG |
| 28S_900 | ACCCGACCCCCCCCGCCCCCGCGCCCCGGGACGGACCCGGGGCGGGGAAC |
| 28S_950 | CTGGAGGCGCGCGCCACACGCGCGGCGCCGCGCCGAGCCGCGCGCCGGAC |
| 28S_1000 | AAGGGGGAAGGTTCCCCCCCGGCGCCCCCCGCGGCCTCGCCCGCGCCGGG |
| 28S_1050 | CCCGTCCCCCGAACGGGCCGCCCCCGCGGGAACGGAGGCGGCCCGAACAG |
| 28S_1100 | CACTGAGCGCAGTCCGCCCCGGTCGGACAGCGGCGCCGGGGGCCGGCGGG |
| 28S_1150 | CGCCCTGGCGTGGCCCGAGCCCGGCCCGGCGGCGCCGCGCGGTCGGGGCG |
| 28S_1200 | GTGTTTCAAGACGGGTCGGGTGGGTAGCCGACGTCGCCGCGGACCCCGGG |
| 28S_1250 | GGGCTTTCGCGCGAGCCGCCGACTCGCGCGCGTGCTAGACTCCTTGGTCC |
| 28S_1300 | GGGATCCCACCTCAGCCGGCGCGCGCCGGCCCTCACCTTCATTGCGCCGC |
| 28S_1350 | CGGGCGAGACGGGCCGGTGGTGCGCCCGGGGCCTTCCGGCCTGCCGCCCC |
| 28S_1400 | CATCTTTCGGGTCCTAGCACGCGCGCTCATGCTCCACCTCCCCGGCGAGG |
| 28S_1450 | GACCTCCACCAGAGTTTCCTCTGGCTTCGCCCTGCCCAGGCATAGTTCAC |
| 28S_1500 | CGCCCCTATACCCGGGTCGGACGACCGATTTGCACGTCAGGACCGCTACG |
| 28S_1550 | TGAGGGAAACTTCGGAGGGAACCAGCTACTAGATGGTTCGATTAGTCTTT |
| 28S_1600 | ATTCGCTTTACCGGGTAAAACTGCACCGCCGCCCCGAGCGCCAGCTATCC |
| 28S_1650 | TGAAAGTTTGAGAATAGGTTGAGATCGTTTCGGCCCCAAGACCTCTAATC |
| 28S_1700 | TCGCATTCCACGGCCCGGCTCCACGCCAGCGAGCCGGGCGTCTTACCCAT |
| 28S_1750 | GTTCATCCCGCAGCGCCAGTTCTGCTTACCAAAAGTGGCCCACTGAGCGC |
| 28S_1800 | TTTCTGGGCTCTGATGAGCGTCGGCATCGGGCGCCTTAACCCGGCGTTCG |
| 28S_1850 | GGTTCCGACTTCCATGGCCACCGTCCTGCTGTCTAGATCAACCAACACCT |
| 28S_1900 | TTTTCAGGGCTAGTTGATTCGGCAGGTGAGTTGTTACACACTCCTTAGCG |
| 28S_1950 | CGCACCGCCGGCGACGGCCGGGTATGGGCCCGACGCTCCAGCGCCATCCA |
| 28S_2000 | GGCGCACCGCAGCGGCCCTCCTACTCGTCGCGGCGTAGCCCCCGCGGCTC |
| 28S_2050 | AAGATCTGCACCTGCGGCGGCTCCACCCGGGCCCGCGCCCCAGGCTTCCA |
| 28S_2100 | GCTCCACTTCGGCCTTCAAAGCTCTCGTTTGAATAGTTGCTACTACCACC |
| 28S_2150 | TCGCTTAGGACCGACTGACCCATGTTCAACTGCTGTTCACATGGAACCCT |
| 28S_2200 | CTGAGGGCAACGGAGGCCATCGCCCGTCCCTTCGGAACGGCGCTCGCCTA |
| 28S_2250 | TCTCCGCCGCTCCGGATTCGGGGATCTGAACCCGACTCCCTTTCGATCGG |
| 28S_2300 | GCTTCTCCGGGATCGCTTGCGTTACCGCACTGGGCGCCTCGCGGCGCCCG |
| 28S_2350 | GCGCCCGGCCCTTCACAAAGAAAAGAGAACTCTCCCCGGGGCTCCCGCCG |
| 28S_2400 | CGACGCTTTCCAAGGCGCGGGCCCCTCTCTCGGGGCGAACCCGTTCCAGG |
| 28S_2450 | CCCCGGATTTTCACGGGCCAGCGAGAGCTCACCGGACGCCGCCGGAACCG |
| 28S_2500 | AGACCTGCTGCGGATATGGGTACGGCCCGGCGCGAGATTTACACCCTCTC |
| 28S_2550 | GACTTCCCTTACCTACATTGGTCCAACATGCCAGAGGCTGTTCACCTTGG |
| 28S_2600 | CCCAGCCCTTAGAGCCAATCCTTATCCCGAAGTTACGGATCCGGCTTGCC |
| 28S_2650 | GTCCAGCCGCGGCGCGCGCCCAGCCCCGCTTCGCGCCCCAGCCCGACCGA |
| 28S_2700 | CCCCGGCGAGCGGGAGCGGGGAAAGGGGGGGCGGGGGCGGGCGGCGCCTC |
| 28S_2750 | CGCGTCCAGAGTCGCCGCCGCCGCGCCGCCCGCTGACCCCCCCCCCGGCG |
| 28S_2800 | GGCGAGCGGCGCCCGCCGCAGCTGGGGCGATCCACGGGAAGGGCCCGGCG |
| 28S_2850 | GGCCGACGGGAGGGGCGCCGGGAGCGGGGGGGCGGAGGGGCAAGGAGGGG |
| 28S_2900 | CTCGCGCCCTCGCGGGGAGGGCGCTCGGGACGGGGGGCGGCCGGGACGAC |
| 28S_2950 | CGGCGGGCCCCCCCCCGCCGCCGCCGCGCCCGCGGCCGCCGCCGCCGCCC |
| 28S_3000 | GCGCCGGCCCGGAGACCCCCCCCGCCCGGGACCGCCCCGCCCGGCGCCGC |
| 28S_3050 | CCTGGTCCGCACCAGTTCTAAGCCGGCTGCTAGGCGCCGGCCGAGGCGGG |
| 28S_3100 | CCCGCCGCGGGCCTTCGCGATGCTTTGTTTTAATTAAACAGTCGGATTCC |
| 28S_3150 | TCTTCACTTTGACATTCAGAGCACTGGGCAGAAATCACATCGCGTCAACA |
| 28S_3200 | CTTAAGAGAGTCATAGTTACTCCCGCCGTTTACCCGCGCTTCATTGAATT |
| 28S_3250 | GTTCATCCATTCATGCGCGTCACTAATTAGATGACGAGGCATTTGGCTAC |
| 28S_3300 | TCCCTTGGCTGTGGTTTCGCTGGAGAGTAGGTAGGGACAGTGGGAATCTC |
| 28S_3350 | GAGTCAAGCTCAACAGGGTCTTCTTTCCCCGCTGATTCCGCCAAGCCCGT |
| 28S_3400 | GGCCTCCCACTTATTCTACACCTCTCATGTCTCTTCACAGCGCCAGACTA |
| 28S_3450 | AGTGGTATTTCACCGGCGGCCGGGCCGCGGCGCGGGTCGCGCGACCGCGG |
| 28S_3500 | TCGGGGCTCGCCCCCCCGCCTCACCGGGTAAGTGAAAAAACGATCAGAGT |
| 28S_3550 | GGGTCGCGCCCGGCGCGCGCCGGGCGCTTGGCGCCAGAAGCGAGAGCCCC |
| 28S_3600 | ACAGGTGTACCGCCCCAGTCAAACTCCCCACCTGACGCTGTCCCCGGAGC |
| 28S_3650 | AGGTTTCTGGCCTCCCTGAGCTCGCCTTAGGACACCTGCGTTACGCTTTG |
| 28S_3700 | TCGTACTGAAAATCAAGATCAAGCGAGCTTTTGCCCTTCTGCTCCACGGG |
| 28S_3750 | AAACCCAAAAAGTCAGAAGGATCGTGAGGCCCCGCTTTCACGGTCTGTAT |
| 28S_3800 | CGCCACAAGCCAGTTATCCCTGTGGTAACTTTTCTGACACCTCCTGCTTA |
| 28S_3850 | AAGAGCCGACATCGAAGGATCAAAAAGCGACGTCGCTATGAACGCTTGGC |
| 28S_3900 | TAGTGGGTGAACAATCCAACGCTTGGTGAATTCTGCTTCACAATGATAGG |
| 28S_3950 | GTAAAACTAACCTGTCTCACGACGGTCTAAACCCAGCTCACGTTCCCTAT |
| 28S_4000 | TCCTCTCGTACTGAGCAGGATTACTAGCGCAACAACACATCATCAGTAGG |
| 28S_4050 | TCCAGTGGCTCCTCAGCCAAGCACATACACCAAATGTCTGAACCTGCGGT |
| 28S_4100 | GATTCTGACTTAGAGGCGTTCAGTCATAATCCCACAGATGGTAGCCTCGC |
| 28S_4150 | GCGAGCCCACCGAGGCGCCTCGGCGCTGCGGTATCGCTACGTTTAGGGGG |
| 28S_4200 | GCGGCGCTCCGCACCGACCGCCCGCCCGAGGGGGGCGGCGGCCGGCTATC |
| 28S_4250 | GCGGGGGAGAGGCGGCGCCACATCTGTCCGCGCTCCGGTCCCGACCACGA |
| 28S_4300 | TCGTCTACGAATGATTTAGCACCGGGTTCCCCACGAACATGCGGTACGCG |
| 28S_4350 | GCAGCGAGGGAGCTGCTCTGCTACGTACGAAACCCCGACCCAGAATCAGG |
| 18S_0 | TCGCAGTTTCACTGTACCGCCCGTGTGTACTTAGACATGCATGGCTTAAT |
| 18S_50 | GTAACGGGAGGGGAGCGACCAAAGGAACCATAACTGATTTAATGAGCCAT |
| 18S_100 | GTCGGCGCTCGTCGGCATGTATTAGCTCTAGAATTACCACAGTTATCCAA |
| 18S_150 | CGGGCGAGCCCGGGTTGGTTTTGGTCTGATAAATGCACGCGTCCCCGGAG |
| 18S_200 | CCACGGGGGCGTGCGATCGGCTCGAGGTTATCTAGAGTCACCAAAGCCGC |
| 18S_250 | ACAGTACCATCGAAAGTTGATAGGGCAGACATTCGAATGGGTCGTCGCCG |
| 18S_300 | GGAATCGAACCCTGATTCCCCGTTACCCGTGGTCACCATGGTAGGCACAG |
| 18S_350 | CGCCTGCTGCCTTCCTTGGATGTGGTAGCCGTTTCTCAGGCTCCCTCTCC |
| 18S_400 | ATTGTTATTTTTCGTCACTACCTCCCCGGGTCGGGAGTGGGTAATTTGCG |
| 18S_450 | AGGATTTAAAGTGGACTCATTCCAATTACAGGGCCTCGAAAGAGTCCTGT |
| 18S_500 | ATTACCGCGGCTGCTGGCACCAGACTTGCCCTCCAATGGATCCTCGTTAA |
| 18S_550 | CTACGAGCTTTTTAACTGCAGCAACTTTAATATACGCTATTGGAGCTGGA |
| 18S_600 | AGGCGGTAGCTCGCCTCGCGGCGGACCGCCAGCTCGATCCCAAGATCCAA |
| 18S_650 | GACACTCAGTTAAGAGCATCGAGGGGGCGCCGAGAGACAGGGGCTGGGAC |
| 18S_700 | CTGCTTTGAACACTCTAATTTTTTCAAAGTAAACGCTTCGGGCCCCGCGG |
| 18S_750 | AACCGGAGTCCTATTCCATTATTCCTAGCTGGAGTATTCCGGCGGCCAGC |
| 18S_800 | CCGGCCGTCCCTCTTAATCATGGCCCCGTTTCCGAAAACCAACAAAATAG |
| 18S_850 | TCTTGCGCCGGTCCAAGAATTTCACCTCTAGCGGCACAATACGAATGCCC |
| 18S_900 | CGTTCTTGATTAATGAAAACATTCTTGGCAAATGCTTTCGCTTTAGTTCG |
| 18S_950 | TTATGGTCGGAACTACGACGGTATCTGATCGTCTTCGAACCTCCGACTTT |
| 18S_1000 | TGCCCGGCGGGTCATGGGAATAACGCCGCCGGATCGCGAGTCGGCATCGT |
| 18S_1050 | TTTGCAACCATACTCCCCCCGGAACCCAAAGACTTGGGTTTCCCGGGAGC |
| 18S_1100 | CAGGCTCCACTCCTGGTGGTGCCCTTCCGTCAATTCCTTTAAGTTTCAGC |
| 18S_1150 | GTCCGTGTCCGGGCCGGGTGAGGTTTCCCGTGTTGAGTCAAATTAAGCCG |
| 18S_1200 | ATGCACCACCACCCACGGAATCGAGAAAGAGCTCTCAATCTGTCAATCCT |
| 18S_1250 | TTCGTTATCGGAATTAACCAGACAAATCGCTCCACCAACTAAGAACGGCC |
| 18S_1300 | GACGCCGACCGCTCGGGGGTCGCGTAACTAGTTAGCATGCCAGAGTCTCG |
| 18S_1350 | TATTGCTCAATCTCGGGTGGCTGAACGCCACTTGTCCCTCTAAGAAGTTG |
| 18S_1400 | CAGTGTAGCGCGCGTGCAGCCCCGGACATCTAAGGGCATCACAGACCTGT |
| 18S_1450 | AACGGGTTACCCGCGCCTGCCGGCGTAGGGTAGACACAAGCTGAGCCAGT |
| 18S_1500 | CGTTCATGGGGAATAATTGCAATCCCCGATCCCCATCACGAATGGGGTTC |
| 18S_1550 | CAGGGACTTAATCAACGCGAGCTTATGACCCGCACTTACTGGGAATTCCT |
| 18S_1600 | CTCACTAAACCATCCAATCGGTAGTAGCGACGGGCGGTGTGTACAAAGGG |
| 18S_1650 | CGACGCTCCGGCAGGGCCGTGGCCGACCCCGCCGGGGCCGATCCGAGGAC |
| 12S_0 | GCATGTATATGTCTAGCAAAAACCAATAGAAAGGTTAGGACTAAGTCTTT |
| 12S_50 | TTGCTTGGGAAGAAAGGTTTGGGGGCATTTTCACTGGGATGCGGATACTT |
| 12S_100 | TAAGCAAGGCGTCTTGGGCTACTGCTGAGTGTGCCTGATACCTGCTCCTT |
| 12S_150 | CACTTATTGCTTAAGGTTAATTACTGCTGAGTACCCGTGGGGGTGTGGCT |
| 12S_200 | GCTGGCACAAGATTTACCAACCCTGGGTTGCTATGGCTAAGTCAAGTTTA |
| 12S_250 | CTCTTTACGCCGGGTAGCTATTGATTTGGGTTTCTTGTATGACCGCGGTG |
| 12S_300 | GACAGCTTGGTTGCATTTTAATCTTAGCTGGTGCAGATAACATGTGGCCA |
| 12S_350 | GTTTAGGCTAAGATGGATTTGGGTTGGGTTTAGGTGGATCTTAGGCTTAT |
| 12S_400 | GGGTATCTAATCCCAGTTTGGGTCCTAGCTTTCGTGGGTTAAAATTAATC |
| 12S_450 | GCGGATACATGTGTGATGGGAGGTATCTAGATTTAGGGCTAGGCATAGTG |
| 12S_500 | GGCACCGCCAAGTCCTTAGAGTTTTAAGCGTTTGTGCTCGTAGTTCTCAG |
| 12S_550 | GTGAATCGTGGATTATCGATTATAGAACAGGCTCCTCTAGGTGGGTTTGG |
| 12S_600 | GGTGGGCTGGCGACGGCGGTATGTAGGCTGTGCTGGCAAGGGGTGGTTGG |
| 12S_650 | CTGTCTTATTAGCGAGGGGCTATTGAGCTCACTGTTGTTCTTTCATTAGA |
| 12S_700 | ATGTTAGAAAATGTAGCCCATTTCTCCCACCCCATAGGCTATACCTTGAC |
| 12S_750 | GCTAAATCCTCCTTCTAAGGGCGGGTTTCACGTCCTTTTTCGTTTGTTCT |
| 12S_800 | GCCTCAGAGCCGTCTTAAAGTGAGCTTAGGGGGTATGATCTCACTTTACT |
| 12S_850 | ATTTATTGATGTTGATGGCTTGTGAAGAGGGTGACGGGCGGTATGTACGT |
| 12S_900 | ACTTACCTTGTTACGACTTGCCTCGTCTTTAGCCGGGAGGGGAGGTATAT |
| 16S_0 | TAAGGGTACGAGTTTGTCGGGCTAGAGGGGGGGCAAGTCAAGGCGACCTT |
| 16S_50 | GGGACAATTTATAATGTTTTGGTTAAGAGGGGGAGGTAAGTTTTTTATGT |
| 16S_100 | GTACAGTTAGCCTCTATTGCGCCGGGGTAGTCTTTTCTATCGCCTATACT |
| 16S_150 | TTGCTGTTTTTTGCTTATGGTTTTCATTGCTATTTCATCTTTCCCTTGCG |
| 16S_200 | GCTTGGTTGTTCTTGCTAAATCATGATGCAAAAGGTACAAGGGTTGGTCT |
| 16S_250 | CTCGCAAGTAGCTCGCTTGGGTTTCGGGAAGGCAAACTTTAGCTCACTTT |
| 16S_300 | AGTCATCCCACTCTTTTGCAACAGAGACGGGTTCGCTCAAATTTTAGCTG |
| 16S_350 | CAGGTAACCAGCTATCACCCAGCTCGGTAGGCTTTTCACCTCTACTGGCA |
| 16S_400 | AGGTGGGTGTCTTTAGGGGGTGGGTTAAGGGGGAACTTAGATTCGTTTGA |
| 16S_450 | CTGTACCCCCATCGAATTGCTCTTAACCCCAACGTTCTCAAGGTTGACAA |
| 16S_500 | GAGGGGTGATTATTATCCGCTGGAGGAGGTTGTGTTCTTTTTCGATGGAG |
| 16S_550 | GCTTTGACGCACTCTTTTGTTGGTGGCTGCTTGAAGGCCCACAGTGCGGG |
| 16S_600 | CTGCTTTGGTTGAGGGAGTCAAATAGGGTTTTAGATTTTTTAATGAGGGA |
| 16S_650 | GTTCCAGATTACTCATTTTAGCATTGATTCTTCTATTGTCATAGGTTAAC |
| 16S_700 | TGAGTTCTGTTAATAATGTATTAATGTAAGTTTACGCCGTAGGAGGATAG |
| 16S_750 | GCTTAACAGATTGAGGAATACGTATTGCTTGTTAGTGTGGGGGTATAAGT |
| 16S_800 | GCCGAGTTCCTTCTGTAGGTTTTAATCATCCTGTGGGCGCTCCTGGGTTG |
| 16S_850 | TGTTGTTAGCTGAAGGCTATGTTTTTGGGAAACAGTCGGGTCTTTGGTTT |
| 16S_900 | CGCGGCCGTTGAACTTTGGGGGTCACTGGGCAGGCATCACCTTCAATACT |
| 16S_950 | TCTCAATTTACGGGACAATTGATTGCGCTACCTTCGCACGGTTAGGATAC |
| 16S_1000 | TAGATTACCTACAGGAGACAGTTAAGACCTCGTTTAGCCATTCATACAAG |
| 16S_1050 | TTCTCGTCTTATGTTCACATTCTCGTTTTTGCACGGGAATACTAATTTCA |
| 16S_1100 | GGGTTGTGTAAGGTTGTAAGGTGGTCGTGATTTTAAAGTTCCACAGGGTC |
| 16S_1150 | CCCCAACCGAAAAATGTCGACCAGGGGTTTATGTGTGGGTGGACCCAGTG |
| 16S_1200 | TGAAGAGTTGTGGTCTGTGGGTTTGGAGGATTTTTTTTTCTCCAAGGTCG |
| 16S_1250 | CAATTATATTGGGTCTGGTTACTGTTGGTACTTTGAGGAGTTGGTCTTAG |
| 16S_1300 | CTCTTGGAGGAGATTGCGCTGTTATCCCTGGGGTAGCTTGGTCCATTGCT |
| 16S_1350 | AGGTTGTCCTGATCCAACATCGAGGTCGTAAACCTCCTTGTCGATATGGG |
| 16S_1400 | AGGACTGTTAATCGTTGAACAAACGAACCCTTAATAGCGGTTGCACCATT |
| 16S_1450 | CCATAGATAGAAACCGACCTGGATTGCTCCGGTCTGAACTCAGATCACGT |
| 16S_1500 | AGTGGTATTGACCCCACTTCTCCGGTCCTTTCGTACTAGGAGGAGTGTGT |
| 16S_1550 | GTTCTTGGCAGTTGAGTTGTATTCATTGCTTAGAAGGTTGGGGTGTGCTC |
| 5S_0 | TTCCGAGATCAGACGAGATCGGGCGTTACCAGGGTGGGATGGCCGTAGGC |
| 5S_50 | TCCCAGGAGGTCTCCCATCCAAGTACTAACCAGGCCCGACCCTGCTTAGC |
