# Supplementary figures and images for: Novel rRNA-depletion methods for total RNA sequencing and ribosome profiling developed for avian species
Source: Poult Sci. 2021 Jun 9;100(9):101321. doi: 10.1016/j.psj.2021.101321 (PMC8322463; doi:10.1016/j.psj.2021.101321)

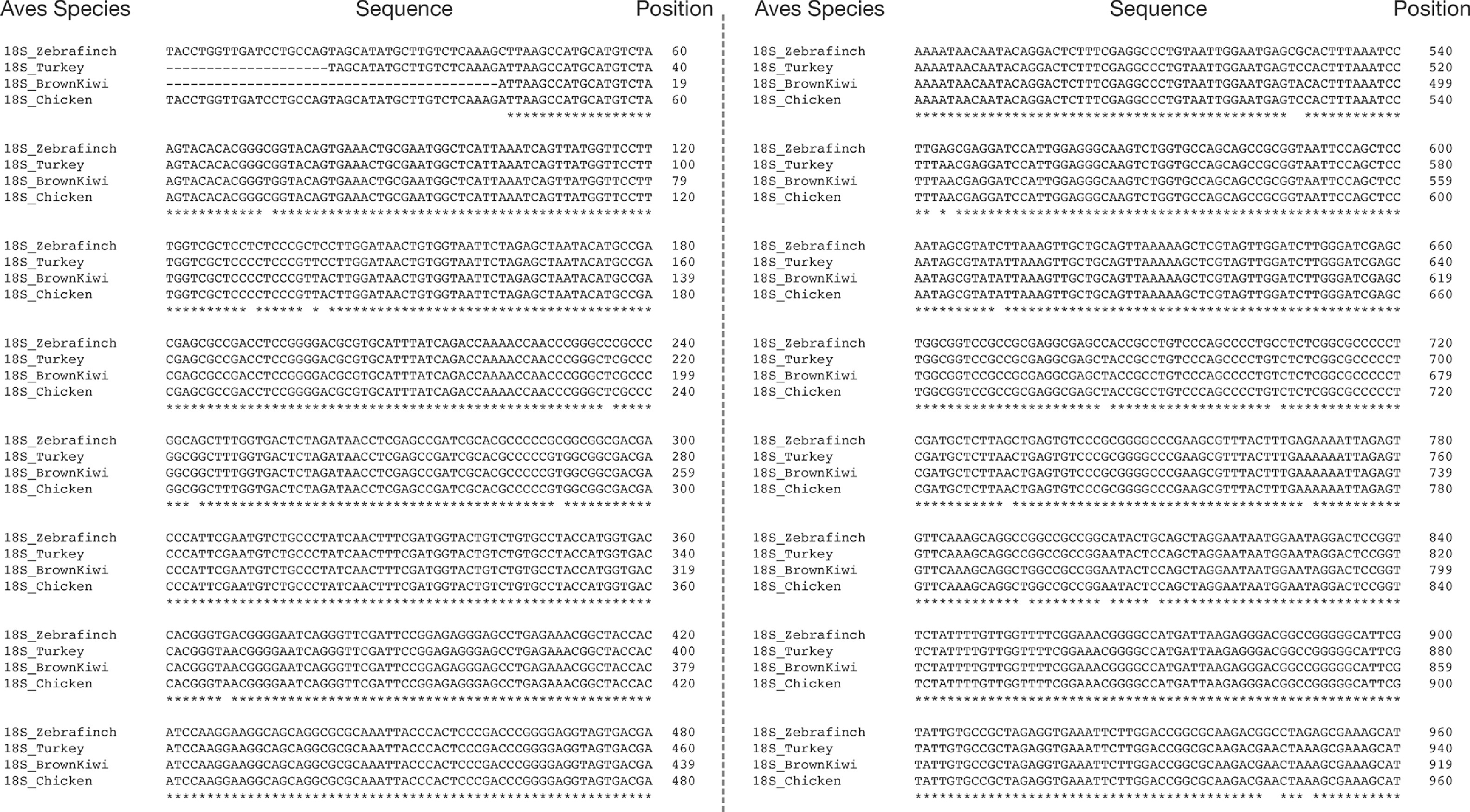

Supplement: Supplementary file 2 [file mmc2.jpg]

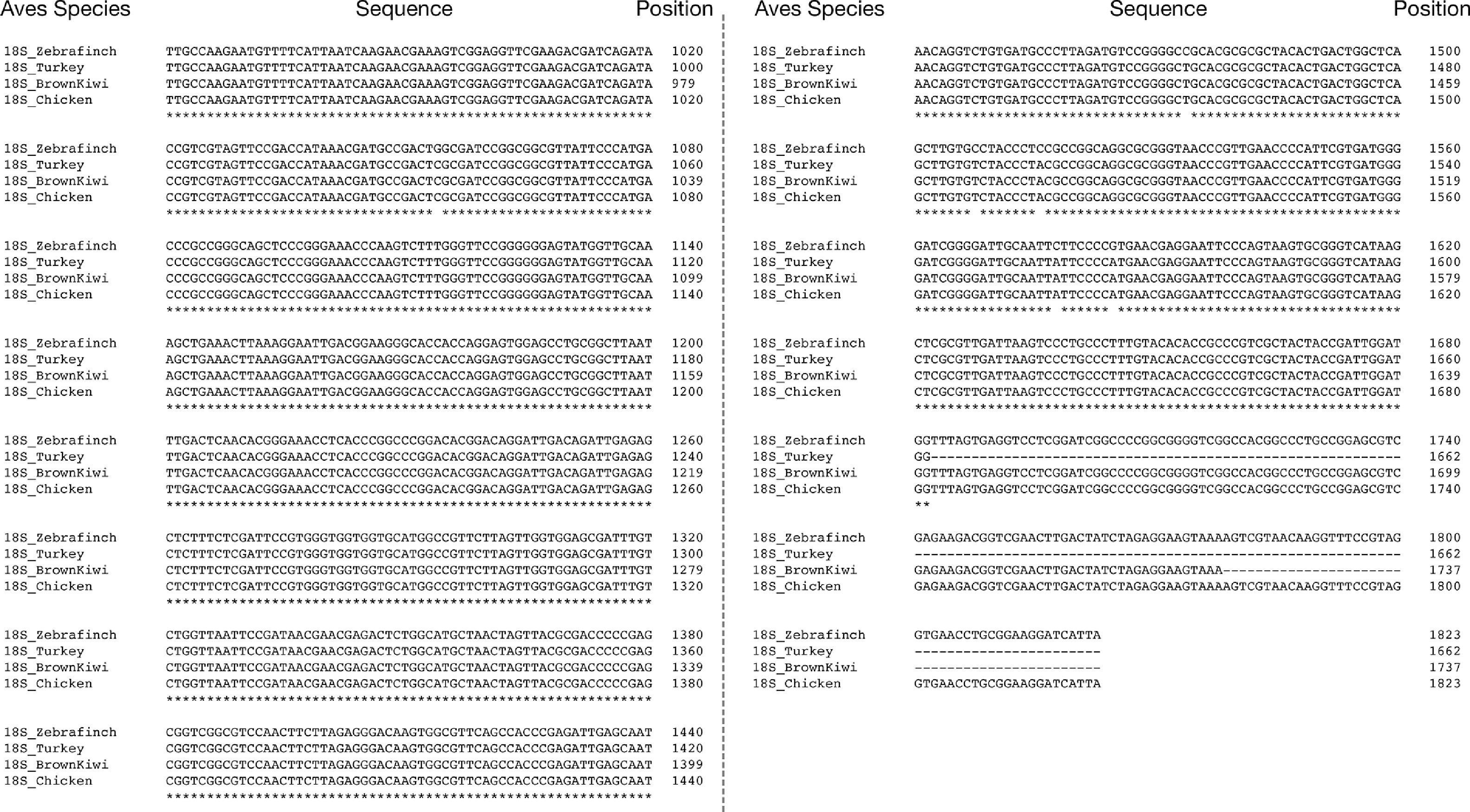

Supplement: Supplementary file 3 — Supplementary Figure 1: Alignment of 18S sequences from four avian species: chicken (Gallus gallus), zebra finch (Taeniopygia guttata), north island brown kiwi (Apteryx mantelli) and turkey (Meleagris gallopavo). [file mmc3.jpg]
